# Clinical characteristics of bloodstream infections in the patients receiving anti-IL-6 receptor antibody

**DOI:** 10.1007/s15010-026-02938-0

**Published:** 2026-08-18

**Authors:** Koh Shinohara, Kentaro Tochitani, Yoshiki Katada, Tatsunori Murata, Mayu Morita, Yusuke Tsuda, Yasuhiro Tsuchido, Masaki Yamamoto, Yasufumi Matsumura, Miki Nagao

**Affiliations:** 1 Department of Clinical Laboratory Medicine, Kyoto University Graduate School of Medicine, 54 Shogoin-Kawahara-cho, Sakyo-ku, Kyoto, Japan; 2 Department of Infection Control and Prevention, Kyoto University Hospital, 54 Shogoin- Kawahara-cho, Sakyo-ku, Kyoto, Japan; 3 Department of Infectious Diseases, Kyoto City Hospital, 1-2 Mibu-Higashi-Takada-cho, Nakagyo-ku, Kyoto, Japan; 4 Department of Clinical Pharmacology and Therapeutics, Kyoto University Hospital, 54 Shogoin-Kawahara-cho, Sakyo-ku, Kyoto, Japan; 5 Department of Pharmacy, Kyoto City Hospital, 1-2 Mibu-Higashi-Takada-cho, Nakagyo- ku, Kyoto, Japan

**Keywords:** Bloodstream infection, Infection, Immunocompromised, Interleukin-6, IL-6, Anti-IL6R antibody, Anti-IL-6 receptor antibody, Sarilumab, Tocilizumab

## Abstract

**Purpose:**

Although indications for anti-interleukin 6 (IL-6) receptor antibody (anti-IL6R) therapy continue to expand, the clinical features of bloodstream infection (BSI) in anti-IL6R recipients remain poorly characterized. We investigated the prevalence, clinical and microbiological features of BSI among the anti-IL6R recipients.

**Methods:**

We conducted a multicenter, retrospective study of BSI episodes occurring within 90 days of anti-IL6R administration between 2005 and 2024. Complicated BSI was defined as the presence of either (1) infection-related mortality, (2) remote or localized suppurative complications, (3) embolic stroke, or (4) recurrence within 90 days. Associations between clinical variables and complicated BSI were analyzed.

**Results:**

Among 922 anti-IL6R recipients, 47 patients (5%) developed BSI. Demographics varied by indication for anti-IL6R, and 89% received concomitant immunosuppressive therapy. Catheter-related bloodstream (23%) and intra-abdominal (21%) were most common foci, followed by bone and joint (14%). At presentation, fever was absent in 53%, and serum CRP was within normal range in 30%. Septic shock occurred in 26%, and all-cause 30-day mortality was 19%. Complicated BSI occurred in 22 episodes (47%). In univariate analysis, underlying rheumatic disease, community acquisition, onset within 21 days of anti-IL6R administration, and *Staphylococcus aureus* or *Klebsiella pneumoniae* etiology were associated with complicated BSI, whereas higher pulse rate at presentation and Pitt bacteremia score ≥ 4 were inversely associated.

**Conclusions:**

In anti-IL6R recipients, BSI frequently lacked typical signs such as fever but often resulted in severe outcomes. Clinicians should maintain a high index of suspicion for complicated BSI in this population.

## Introduction

Interleukin-6 (IL-6) is a pleiotropic cytokine with a pivotal role in both innate and adaptive immunity. Excessive production of IL-6 and dysregulation of its signaling pathway contribute to the pathogenesis of various immune-mediated diseases [1, 2]. Consequently, molecular-targeted drugs that inhibit the IL-6 signaling pathway have been developed and the indications of anti-IL-6 agents have expanded to include rheumatic and autoimmune disorders such as rheumatoid arthritis (RA) and multicentric Castleman disease (MCD), severe COVID-19, and cytokine release syndrome (CRS) associated with immune-related therapies such as immune checkpoint inhibitors or chimeric antigen receptor T-cell (CAR-T) therapy [2]. In US, anti-IL-6 receptor antibodies (anti-IL6R Ab, tocilizumab and sarilumab), anti-IL-6 monoclonal antibody (siltuximab), and pH-dependent anti-IL6R antibody (satralizumab) have been approved by the Food and Drug Administration (FDA) for clinical use. In Japan, as of 2024, two anti-IL6R Ab, tocilizumab and sarilumab, are approved for indications including RA (tocilizumab and sarilumab), Takayasu’s arteritis, giant cell arteritis, juvenile idiopathic arthritis, adult-onset Still’s disease, MCD, CRS, and severe COVID-19 (tocilizumab).

Nevertheless, blockade of IL-6 pathway influences host control of infections. In patients with anti-IL6R Ab, cases of recurrent or disseminated *Staphylococcus aureus* subcutaneous abscesses [3, 4], and empyema caused by *Escherichia coli* and *Streptococcus intermedius* [4] have been reported. Among RA patients, use of anti-IL6R therapy - primarily tocilizumab - has been associated with serious bacterial infections, skin and soft tissue infections, and diverticulitis [5, 6]. Moreover, multiple cases of disseminated *S. aureus* infections were reported in patients treated with tocilizumab [7–9]. Previous studies have also suggested that the clinical presentations of infections in patients receiving anti-IL6R Ab are often atypical, such as mild systemic and local inflammatory signs despite disseminated infection, or having normal-to-low C-reactive protein (CRP) levels [10, 11]. Because IL-6 is a major stimulus for CRP synthesis in the liver as a part of acute-phase response during infection [12], the inhibition of IL-6 leads to a suppression of CRP production, which can impede the diagnosis of infections and thus increasing the risk of complications [13].

These findings indicate that presentations of severe infections including bloodstream infection (BSI) are likely to be atypical, and complicated BSI including disseminated infections and local suppurative complications would be more problematic in patients receivinganti-IL6R Ab. However, despite widening use of anti-IL6R Abin clinical settings, the clinical features of BSI in patients receiving anti-IL6R Ab have been poorly investigated. To investigate the clinical features of BSI in patients receiving anti-IL6R Ab, especially complicated BSI including disseminated infections, we conducted a multicenter, retrospective cohort study in Japan.

## Methods

### Study design and patients

This study was conducted at two major hospitals in Kyoto, Kyoto University Hospital and Kyoto City Hospital, which have a total of 1679 beds. We identified the patients receiving anti-IL6R Ab by using the pharmacy database and the results of corresponding patients from the microbiological database in each hospital. We screened all episodes with positive blood cultures which sampled within 90 days of administration of anti-IL6R Ab, tocilizumab or sarilumab, from 1 January 2005 through 31 December 2024. Episodes considered as contamination were excluded, following the definition described below. We included all the remaining bacteremia episodes and retrospectively reviewed their medical records. Demographics and clinical courses of all episodes were investigated, and if the multiple episodes in a patient were observed, data of the initial episode was included for the analysis.

## Definitions

Contamination of blood culture was defined as if common contaminant organisms (e.g., coagulase negative staphylococci (CNS); or *Bacillus* spp.; or *Corynebacterium* spp.; or *Propionobacterium* spp.; or *Aerococcus* spp.; or *Micrococcus* spp.) from a single set of the multiple sets of blood culture [14]. We classified the indications of anti-IL6R into four categories: (1) rheumatic diseases, (2) MCD, (3) CRS, and (4) COVID-19. Fever was defined as a temperature superior to 38 °C. Infectious foci were classified and ranked as follows: infective endocarditis and other endovascular infections, bone and joint infections, respiratory tract infections, skin and soft-tissue infections, urinary tract infections, catheter related blood stream infections (CRBSI), undetermined focus, and others. If the multiple foci were identified, the representative infectious focus was determined following the ranking of infectious foci. Complicated BSI were defined as the presence of either (1) infection-related mortality, (2) complicated infections, (3) embolic stroke, or (4) recurrent infections within 90 days [15, 16]. Infection-related mortality was defined as death from direct sequelae of the infection (sepsis, embolic stroke, death during infection control surgery) or persistent signs of infection at the time of death, following the definition in the previous study [17]. Complicated infections were classified into two categories: (i) disseminated complications such as infective endocarditis (IE) or multiple remote infectious foci suggesting hematogenous spreads, and (ii) local suppurative infections like deep seated abscess or thrombophlebitis, and bone and joint infections. The diagnosis of IE was based on the modified Duke Criteria [18]. Sepsis was defined if the case had quick SOFA (qSOFA) score ≥ 2 points, and septic shock was defined if the cases with sepsis and hypotension and hyperlactatemia (lactate > 2 mmol/L) requiring catecholamine [19]. Place of acquisition (community-acquired, healthcare-associated and hospital-acquired) was followed by the criteria by Friedman [20].

### Patients consent statement

This study was approved by the Ethics Committee of the Kyoto University Graduate School and Faculty of Medicine (approval no. R3240 and R5086). The informed consent was waived due to the retrospective design. This study was conducted in accordance with the principles of the Declaration of Helsinki.

### Statistical analysis

Clinical features of BSI in patients receiving anti-IL6R Ab were reported using the appropriate descriptive statistics. For comparing the variables between complicated and uncomplicated BSIs, Fisher’s exact test and Chi-square test were used for categorical variables, as appropriate, and Mann-Whitney U test was used for continuous variables. For assessing the relationship between risk factors and complicated infections, univariate logistic regression was used. A two-sided p-value of < 0.05 was considered to indicate statistical significance. All statistical analyses were performed using the R software version 4.5.1 (R Core Team (2025). R: A Language and Environment for Statistical Computing. R Foundation for Statistical Computing, Vienna, Austria.; https://www.r-project.org). When data were missing, those cases were excluded from the analysis.

This study is reported following the STROBE guidelines for reporting of observational studies [21].

## Results

### Inclusion

**Fig. 1:**
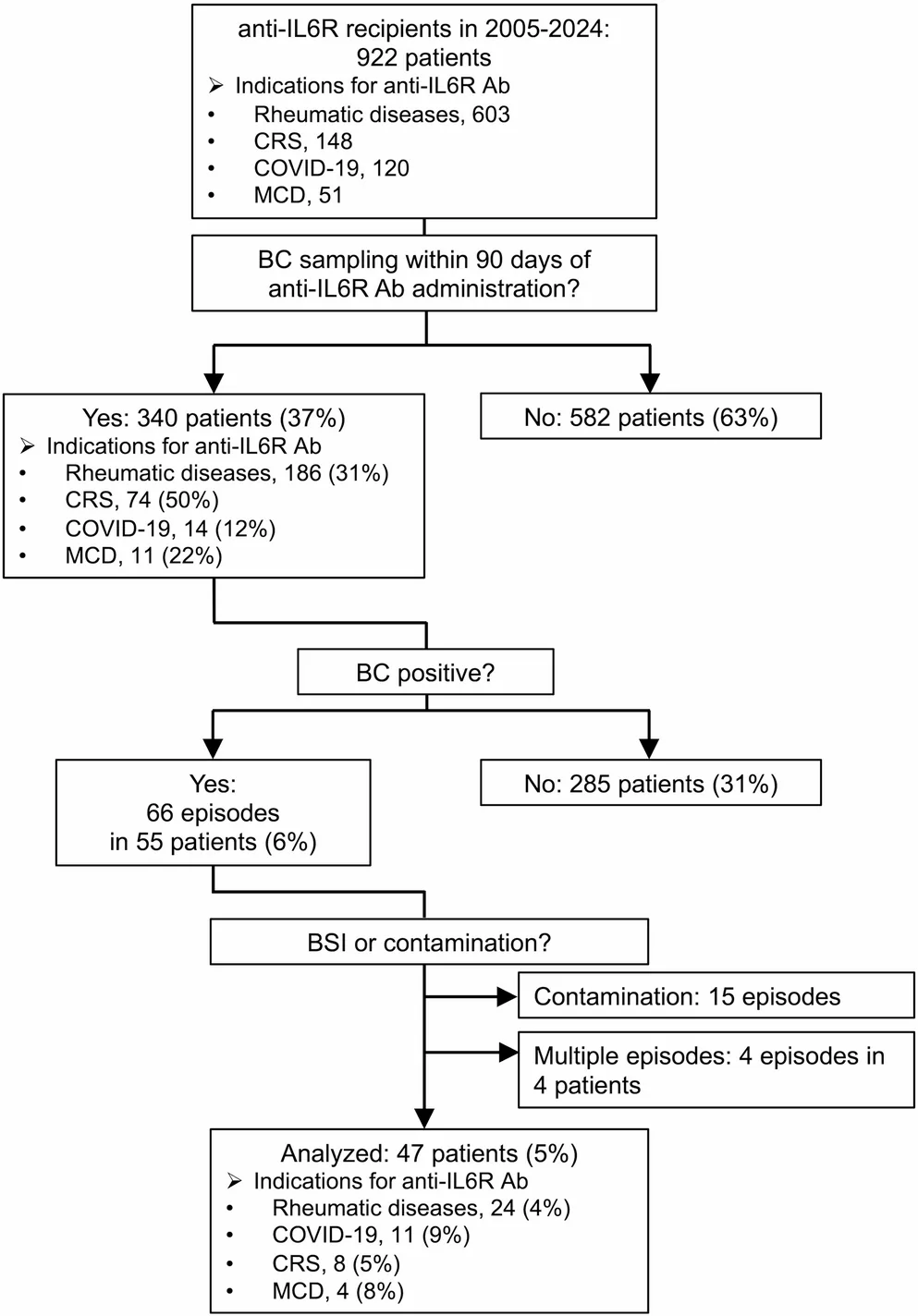
Study flowchart depicting inclusion of patients. anti-IL6R Ab, anti-interleukin-6 receptor antibody, CRS, cytokine-releasing syndrome; MCD, multicentric Castleman disease; BC, blood culture; BSI, bloodstream infection

A total of 922 patients received at least one administration of anti-IL6R Ab during the study period. Blood cultures were obtained within 90 days of anti-IL6R Ab administration in 340 patients (37%). Among them, 66 blood culture positive episodes were observed in 55 patients (6%), and 15 episodes were regarded as contamination and excluded. In 4 patients, two BSIs were observed during the study period, and the first episodes were included for the study. Consequently, 47 episodes in 47 patients (5%) were included in the analysis (Fig. 1).

### Demographics

Demographic features of the 47 patients are shown in Table 1. Median patient age was 70 years, and 22 (47%) were male. Anti-IL6R Ab was most often used for rheumatic diseases (51%), followed by COVID-19 (23%), CRS (17%), and MCD (9%). Among the 24 cases with rheumatic diseases, rheumatic arthritis is the commonest (18 cases), followed by adult-onset Still’s disease (3 cases), juvenile idiopathic arthritis (2 cases). In patients with rheumatic diseases or MCD, most received continuous anti-IL6R Ab treatment, while five patients received only one or two doses because of adverse events or poor efficacy. Of 28 patients with rheumatic disease or MCD, disease activity was stably suppressed prior to the BSI onset in 23 patients (82%). In contrast, all COVID-19 and CRS patients were administered anti-IL6R Ab temporarily. Additional immunosuppressive agents were given to 42 patients (89%); corticosteroids were used in 35 (74%), either chronically (20, 43%; mainly rheumatic diseases or MCD) or as high-dose pulses (20, 43%; mainly COVID-19 or CRS). Among 16 patients with immunosuppressants, methotrexate was most frequently used (seven patients), followed by tacrolimus (four patients) and cyclophosphamide (three patients); six of these patients received two or more immunosuppressants concurrently. Biological agents were administered in eight patients (17%), three of whom received rituximab. Seven patients received additional immunosuppressive therapy other than corticosteroids, including immunosuppressants (*n* = 4), chemotherapy (*n* = 2), and rituximab (*n* = 1). Twelve patients (26%) received at least two concomitant immunosuppressive agents with anti-IL6R Ab. Among the eight patients in the CRS group, CRS was induced by CAR-T therapy in six patients, immune checkpoint inhibitor therapy in one, and bispecific T cell engager therapy in one. Hematological malignancies are the most frequent indications for these therapies, including lymphoid neoplasms in five patients and multiple myeloma in two patients. All six patients who received CAR-T therapy had prior cytotoxic chemotherapy, including rituximab in two patients, and four underwent lymphodepletion therapy with fludarabine with or without cyclophosphamide. No patients had a history of injection drug use or human immunodeficiency virus infection. The median interval between last administration of anti-IL6R Ab and blood culture sampling was 12 days (IQR: 6–23), and 35 episodes (74%) occurred within 21 days of the last dose. Among them, 11 community-acquired, 11 healthcare-associated, and 26 hospital-acquired BSI (21 on wards, 5 in intensive care units [ICUs]) were included. Community-acquired episodes occurred exclusively in rheumatic patients. Indwelling prosthetic materials were inserted in 21 episodes (45%).

**Table 1:** Demographic and clinical characteristics of the cases according to the indications for anti-interleukin-6 receptor antibody (*n* = 47)

Demographical characteristics of patients	Total *n* = 47	Rheumatic *n* = 24	COVID-19 *n* = 11	CRS *n* = 8	MCD *n* = 4	*p*-value, Rheumatic/MCD vs. COVID-19/CRS
Age, y (IQR)	70 (59–76)	70 (58–79)	74 (65–77)	67 (53–73)	63 (56–67)	0.544
Sex, male	22	6 (25)	7 (64)	6 (75)	3 (75)	0.019
Underlying diseases						
Charlson comorbidity index	2 (1–4)	2 (1–2)	1 (1–5)	3 (2–5)	4 (3–5)	0.622
Diabetes	14 (30)	6 (25)	6 (55)	0	2 (50)	1.000
Chronic kidney disease	7 (15)	4 (17)	1 (9)	1 (13)	1 (25)	0.684
Hemodialysis	3 (6)	1 (4)	1 (9)	0	1 (25)	1.000
Liver cirrhosis	3 (6)	0	2 (18)	0	1 (25)	0.557
Solid organ tumor	6 (13)	2 (8)	2 (18)	1 (13)	1 (25)	0.674
Hematological disorder	7 (15)	0	0	7 (88)	0	< 0.001
Collagen disease	24 (51)	24 (100)	0	0	0	< 0.001
Immunosuppressive therapy, any	42 (89)	20 (83)	11 (100)	8 (100)	3 (75)	0.072
Cytotoxic chemotherapy	10 (21)	1 (4)	0	8 (100)	1 (25)	0.009
Corticosteroid	35 (74)	16 (67)	11 (100)	6 (75)	2 (50)	0.087
High-dose, pulse therapy	20 (43)	2 (8)	11 (100)	6 (75)	1 (25)	< 0.001
Chronic use of steroid	20 (43)	16 (67)	1 (9)	1 (13)	2 (50)	< 0.001
PSL equivalent dose of steroid, mg, median (range)	7.75 (2.5–70.0)	7.25 (3–70.0)	15	10	19.8 (2.5–37.0)	0.448
Immunosuppressants	16 (34)	14 (58)	1 (9)	1 (13)	0	0.006
Other biological agents	8 (17)	1 (4)	1 (9)	6 (75)	0	0.005
CAR-T therapy recipient	6 (13)	0	0	6 (75)	0	0.003
Place of acquisition						
Community acquired	10 (21)	10 (42)	0	0	0	0.003
Healthcare associated	11 (23)	8 (33)	0	2 (25)	1 (25)	0.159
Hospital acquired	26 (55)	6 (25)	11 (100)	6 (75)	3 (75)	< 0.001
Any prosthetic material	21 (45)	8 (33)	7 (64)	4 (50)	2 (50)	0.151
CVC including PICC	12 (26)	2 (8)	6 (55)	3 (38)	1 (25)	0.007
Prosthetic joint/spinal implant	8 (17)	6 (25)	2 (18)	0	0	0.445
CV port	4 (9)	1 (4)	0	2 (25)	1 (25)	1.000
Implanted cardiac device/artificial vessel	2 (4)	1 (4)	0	1 (13)	0	1.000
Duration between last anti-IL6R Ab and BSI onset, day (IQR)	12 (6–23)	8 (6–16)	10 (5–21)	25 (12–61)	31 (13–48)	0.250
4 to 21 days of last anti-IL6R Ab	29 (62)	16 (67)	8 (73)	3 (38)	2 (50)	0.763
Indication of blood culture sampling: fever	32 (68)	13 (54)	8 (73)	8 (100)	3 (75)	0.063
Presentation at onset						
Fever (> 38 °C)	22 (47)	7 (29)	7 (64)	5 (63)	3 (75)	0.081
Leukocyte count (/µL), (IQR)	7.2 (3.0-12.3)	9.5 (3.5–12.8)	9.1 (7.9–20.9)	0.6 (0.4–2.8)	3.8 (3.1–6.3)	0.329
Platelet count (/µL), (IQR)	12.5 (1.4–64.0)	16.8 (11.1–22.6)	13.4 (10.5–15.4)	3.0 (1.8–8.1)	7.9 (6.8–12.6)	0.059
C-reactive protein (mg/L), (IQR)	27.2 (2.3–64.0)	34.1 (1.0–76.0)	13.1 (3.1–31.1)	23.2 (10.6–66.2)	19.0 (7.5–51.5)	0.795
Sepsis	22 (47)	8 (33)	9 (82)	3 (38)	2 (50)	0.151
Septic shock	12 (26)	3 (13)	5 (45)	2 (25)	2 (50)	0.182
Major causative organism						
*Staphylococcus aureus*	10 (21)	5 (21)	4 (36)	1 (13)	0	0.496
*Escherichia coli*	8 (17)	5 (21)	1 (9)	2 (25)	0	1.000
Coagulase-negative Staphylococci	4 (9)	1 (4)	2 (18)	1 (13)	0	
*Klebsiella pneumoniae*	4 (9)	2 (8)	1 (9)	0	1 (25)	0.289
*Pseudomonas aeruginosa*	4 (9)	0	1 (9)	1 (13)	2 (50)	1.000
Polymicrobial	3 (6)	1 (4)	0	2 (25)	0	0.557
Infectious foci						
Catheter-related bloodstream	11 (23)	1 (4)	5 (45)	4 (50)	1 (25)	
Intraabdominal	10 (21)	6 (25)	1 (9)	1 (13)	2 (50)	0.168
Bone and Joint	7 (15)	7 (29)	0	0	0	0.031
Respiratory tract	6 (13)	2 (8)	3 (27)	1 (13)	0	0.204
Urinary tract	6 (13)	4 (17)	1 (9)	1 (13)	0	1.000
Infective endocarditis	2 (4)	2 (8)	0	0	0	0.508
Skin and soft tissue	1 (2)	0	0	0	1 (25)	1.000
Undetermined	4 (9)	2 (8)	1 (9)	1 (13)	0	1.000
Complicated BSI	22 (47)	15 (63)	4 (36)	2 (25)	1 (25)	0.136
Clinical course and outcome						
Delayed appropriate treatment > 48 h	2 (4)	2 (8)	0	0	0	0.508
Lacked fever during course	12 (26)	8 (33)	3 (27)	0	1 (25)	0.310
Persistent fever at 72 h	8 (17)	2 (8)	4 (36)	1 (13)	1 (25)	0.240
Positive follow-up Blood culture (per cases performed)	9/29 (31)	6/14 (43)	1/9 (11)	2/5 (40)	0/1	0.720
New ICU admission (exclude ICU-onset cases)	10/42 (24)	4 (17)	3/5 (60)	2 (25)	1 (25)	0.259
Surgical procedure	17 (36)	13 (54)	0	2 (25)	2 (50)	0.005
Infection-related mortality	5 (11)	1 (4)	2 (18)	1 (13)	1 (25)	0.381
30-day all-cause mortality	9 (19)	2 (8)	4 (36)	1 (13)	2 (50)	0.453

IQR, interquartile range; Anti-IL6R Ab, anti-interleukin-6 receptor antibody; CRS, cytokine-releasing syndrome; MCD, multicentric Castleman disease; CAR, chimeric antigen receptor; CVC, central venous catheter; PICC, peripherally inserted central catheter; CV, central venous; BSI, bloodstream infection; ICU, intensive care unit

### Clinical presentations

**Fig. 2:**
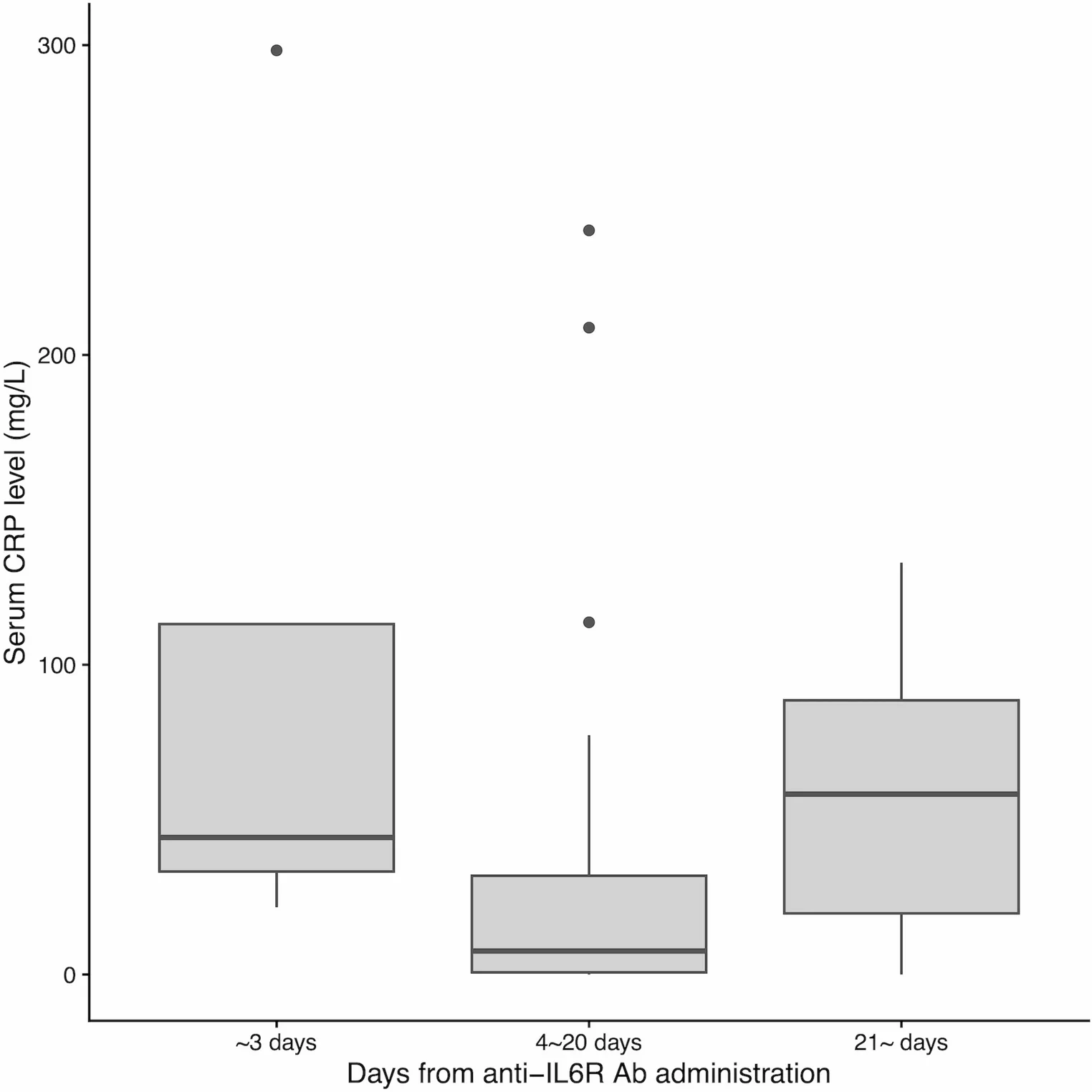
A boxplot depicting serum C-reactive protein level according to the duration between last anti-interleukin-6 receptor antibody (anti-IL6R Ab) administration and blood culture sampling

At the time of blood culture, fever was documented in 22 of 47 episodes (47%). Fever was the indication for blood culture sampling in 32 episodes (68%), including low-grade fever or a recent history of fever before outpatient visits. In the remaining 15 episodes (32%) blood cultures were performed by other findings; localized symptoms suggestive of deep-seated infection (7 episodes), abnormal vital signs or laboratory results (6 episodes), or other reasons such as infectious disease physician recommendation (3 episodes). Serum CRP level at blood culture sampling was within normal limit (< 3.0 mg/L) in 14 episodes (30%). CRP level at blood culture sampling was significantly lower in the episodes occurring 4–20 days after the last anti-IL6R administration than in those within 3 days or after 21 days (median 7.6 mg/L vs. 54.4 mg/L, p-value = 0.005; Fig. 2). Fever was absent throughout the clinical course in 12 episodes (26%). Among 35 febrile episodes, persistent fever > 72 h despite appropriate antimicrobial therapy occurred in 8 episodes (23%). The maximum CRP level within 4 days of onset remained < 100 g/dL in 31 episodes (66%).

### Infectious foci, causative organisms and clinical course

**Fig. 3:**
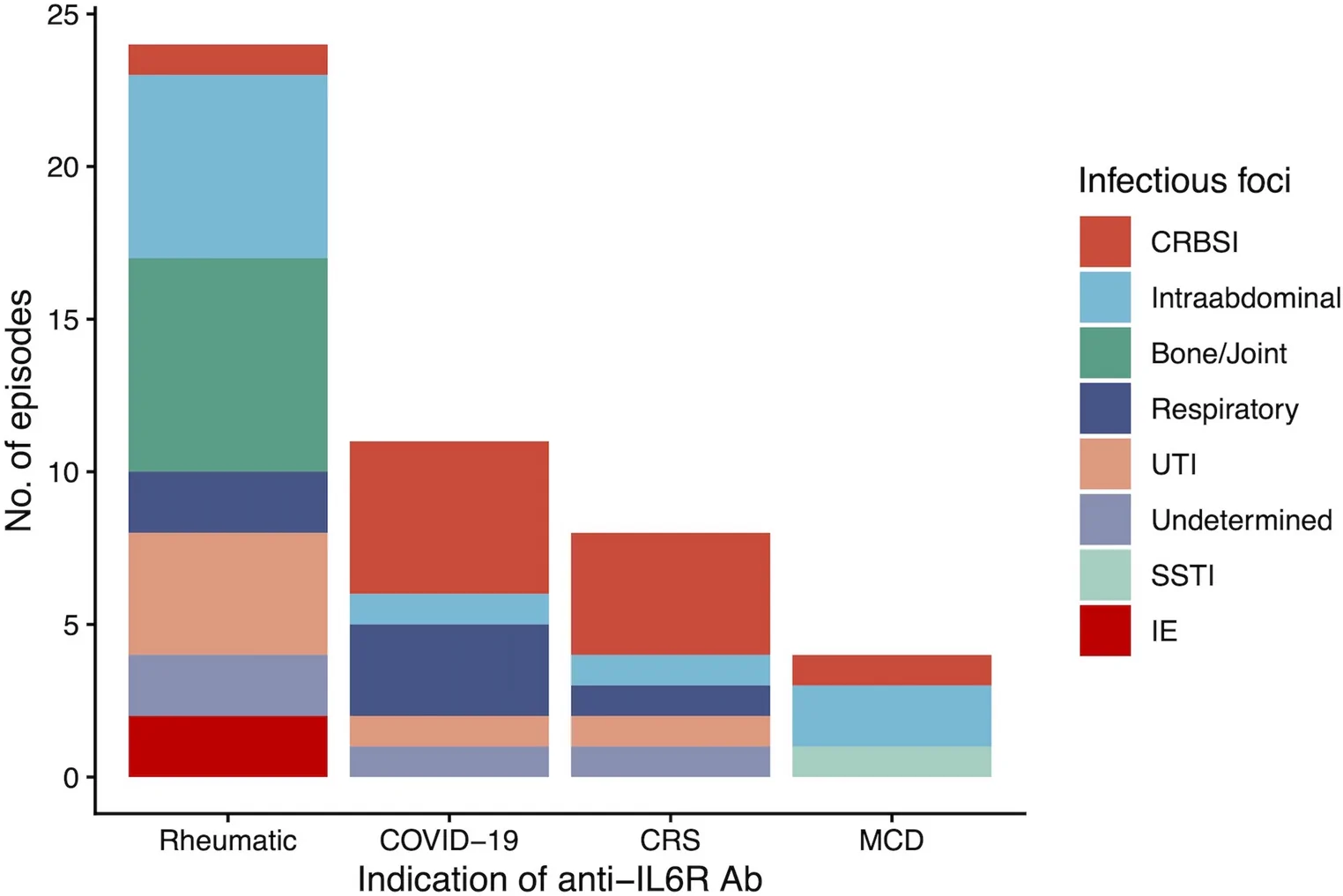
A bar plot depicting distribution of infectious foci according to the indication of anti-interleukin-6 receptor antibody (anti-IL6R Ab)

The most frequent infection site was CRBSI (11, 23%), followed by intra-abdominal (10 episodes, 21%), bone and joint (7, 15%), respiratory, and urinary tract (6 each, 12%). Intra-abdominal episodes included 4 perforative peritonitis, 2 due to diverticulitis. Distribution varied by indication of anti-IL6R Ab; IE and bone and joint infections were confined to rheumatic patients, with only one CRBSI episode (4%), whereas CRBSI predominated in COVID-19 (45%) and CRS (50%) patients (Fig. 3). Place of acquisition also correlated with infectious foci: all community-acquired episodes were either IE (2 episodes), bone and joint infections (5 episodes), or intra-abdominal infections (4 episodes). Most BSIs were monomicrobial (44/47, 94%): Gram-positive organisms in 23 (49%) and Gram-negatives in 21 (45%). *S. aureus* was the most common, followed by *E.coli*, CNS, *Klebsiella pneumoniae* and *Pseudomonas aeruginosa*. No mycobacteria or fungi were detected. Polymicrobial BSIs (3 episodes, 6%) included two CRBSIs (CNS with *E. coli* or *P. aeruginosa*) and one mixed infection (*E. coli* and *Bacteroides fragilis* originated from perforative peritonitis). Methicillin-resistant *S. aureus* (MRSA) was isolated in 4 episodes, and third-generation cephalosporin-resistant Enterobacterales in 2. Follow-up blood culture was obtained in 29 episodes (62%), more often in episodes with Gram-positive bactereia than in those with Gram-negative bacteria (87% [20/23] vs. 33% [7/21], *p* < 0.001). Nine follow-up cultures were positive in 9/29 episodes (31%), including 2 polymicrobial, 2 Gram-negative and 5 Gram-positive episodes, 4 of which were *S. aureus* episodes.

Sepsis occurred in 22 cases (47%), and twelve of them (55%) had septic shock. Septic shock occurred most often with intra-abdominal infections (5/12, including 3 perforative peritonitis). *S. aureus* and *E. coli* each accounted for 3 septic shock episodes. Among septic shock episodes, fever was absent at presentation in 7 episodes (58%) and CRP was normal in 4 (33%). ICU admission was required in 10 of 42 episodes (24%) that onset outside ICU. Surgical intervention was performed in 17 episodes (36%), including operative irrigation and debridement (10 episodes), endoscopic biliary drainage (2), CT-guided drainage (2), central venous port removal (2), and ureter stent insertion (1). In-hospital and 30-day all-cause mortality were 26% and 19%, respectively.

### Complicated BSI and risk factors

Complicated BSI was identified in 22 episodes (47%), including disseminated infections like IE (8 episodes), local complications (13), septic emboli (1), recurrent BSI (4), and infection-related mortality (5), with some overlap (Table 2). Two IE episodes were caused by *Streptococcus gallolyticus* and *Gemella haemolysans*. Local complications included 7 deep-seated abscesses (lung or pleural space 3, soft tissue 2, urinary tract 1, liver 1), 7 bone and joint infections, and 1 thrombophlebitis were documented, including duplicates. Among the 4 recurrent BSI, 2 were *S. aureus* bone and joint infections, and the others were a renal abscess and recurrent cholangitis due to *K. pneumoniae*. Surgical intervention was performed in 9 episodes (41%). In-hospital and 30-day all-cause mortality were not significantly different between complicated episodes (27% and 23%, respectively) and uncomplicated episodes (20% and 16%).

**Table 2:** Details of complicated BSI according to the causative organisms and infectious foci

Organisms	Complicated (*n* = 22)						Uncomplicated (*n* = 25)
		Disseminated	Local complications	Septic emboli	Recurrence	Infection-related mortality	
Gram positive (GP)	12 (52)	6 (26)	6 (26)	1 (4)	2 (9)	5 (22)	11 (48)
*S. aureus*	7 (70)	4 (40)	4 (40)	0	2 (20)	2 (20)	3 (30)
CNS	1 (25)	0	1 (25)	0	0	1 (25)	3 (75)
Other GP	4 (44)	2 (22)	1 (11)	1 (11)	0	1 (11)	5 (56)
Gram negative (GN)	10 (48)	2 (10)	7 (33)	0	2 (10)	2 (10)	11 (52)
*E. coli*	2 (25)	0	1 (13)	0	0	1 (13)	6 (75)
*K. pneumoniae*	3 (75)	0	2 (50)	0	2 (50)	1 (25)	1 (25)
*P. aeruginosa*	2 (50)	1 (25)	2 (50)	0	0	0	2 (50)
Other GN	3 (60)	1 (20)	2 (40)	0	0	0	2 (40)
Polymicrobial	0	0	0	0	0	0	3 (100)
Focus							
Catheter-related Bloodstream	1 (9)	1 (9)	0	0	0	0	10 (91)
Intra-abdominal	4 (40)	2 (20)	1 (10)	0	1 (10)	1 (10)	6 (60)
Bone and Joint	7 (100)	2 (29)	7 (100)	0	2 (29)	0	0
Respiratory tract	4 (67)	0	3 (50)	0	0	2 (33)	2 (33)
Urinary tract	3 (50)	0	2 (33)	0	1 (17)	1 (17)	3 (50)
Undetermined/primary	1 (25)	0	0	0	0	1 (25)	3 (75)
Skin and soft tissue	1 (50)	0	1 (50)	0	0	0	1 (50)
Infective endocarditis	2 (100)	2 (100)	0	1 (50)	0	0	0

CNS, coagulase-negative staphylococci

Underlying rheumatic disease (odds ratio [OR] 3.81 [95% confidence interval [CI] 1.17–13.5], *p* = 0.031), onset within 21 days of anti-IL6R Ab administration (OR 4.98 [95% CI 1.28–25.2], *p* = 0.030), community-acquisition (OR 16.6 [95% CI 2.69–324.], *p* = 0.011), BSI due to *S. aureus* or *K. pneumoniae* (OR 4.38 [95% CI 1.19–18.9], *p* = 0.033) and no other immunosuppressive therapy (OR Inf [1.16-Inf], *p* = 0.017) were significantly associated with the development complicated BSI in univariate analysis (Table 3). Although not significant, persistent positive blood culture appeared to be more frequent in complicated than in uncomplicated BSI (47% [7/15] vs. 7% [2/14], *p* = 0.053). In contrast, Pitt bacteremia score (PBS) ≥ 4 points (OR 0.10 [95% 0.01–0.63], *p* = 0.039) and higher pulse rate at presentation (per /min, OR 0.96 [95% CI 0.93–0.99], *p* = 0.011) were negatively associated with complicated BSI.

**Table 3:** Univariate analysis of risk factors for complicated BSI

Risk factors	Complicated n (%), *N* = 22	Uncomplicated n (%), *N* = 25	Unadjusted OR (95% CI)	*p*-value
Demographics				
Age in year [IQR]	73 [66–81]	63 [58–73]	1.03 (1.00-1.08)	0.086
Male Sex	9 (41)	13 (52)	0.64 (0.20–2.02)	0.448
Days from anti-IL6R Ab administration to BSI onset [IQR]	8 [4–13]	15 [11–28]	0.96 (0.91–0.99)	0.051
BSI onset within 21 days of anti-IL6R administration	19 (86)	16 (64)	4.98 (1.28–25.2)	0.030*
Diabetes	7 (32)	7 (28)	1.20 (0.34–4.27)	0.775
Solid organ tumor	1 (5)	5 (20)	0.19 (0.01–1.32)	0.145
Hematological malignancy	2 (9)	5 (20)	0.40 (0.05–2.10)	0.306
Rheumatic diseases	15 (68)	9 (36)	3.81 (1.17–13.5)	0.031*
Charlson comorbidity score, per point [IQR]	2 [1–3]	2 [1–4]	0.88 (0.63–1.17)	0.386
Indwelling prosthetic materials	9 (41)	12 (48)	0.75 (0.23–2.38)	0.626
Cytotoxic chemotherapy	2 (9)	8 (32)	0.21 (0.03–0.99)	0.071
Corticosteroid use, any	14 (64)	21 (82)	0.33 (0.08–1.27)	0.118
High dose pulse	6 (27)	14 (56)	0.29 (0.08–0.97)	0.051
Chronic use	10 (45)	10 (40)	1.25 (0.39–4.04)	0.706
PSL equivalent dose of steroid, mg, median [IQR]	7.5 [5.0–15.0], *n* = 10	7.75 [5.5–23.5], *n* = 10	0.99 (0.39–1.04)	0.653
Immunosuppressant	7 (32)	9 (36)	0.83 (0.24–2.79)	0.763
No other immunosuppressive therapy	5 (100)	0 (0)	Inf	0.017*
Community acquisition	9 (41)	1 (4)	16.6 (2.69–324)	0.011*
At presentation				
Body temperature > 38 °C	7 (32)	15 (60)	0.31 (0.09–1.01)	0.057
Minimum systolic blood pressure, per mmHg [IQR]	110 [83–131]	93 [79–100]	1.02 (1.00-1.05)	0.055
Maximum pulse rate, per /min [IQR]	92 [81–110]	112 [97–131]	0.96 (0.93–0.99)	0.011*
PBS ≥ 4 points	1 (5)	8 (32)	0.10 (0.01–0.63)	0.039*
Septic shock	4 (18)	8 (32)	0.47 (0.11–1.79)	0.283
WBC count, per 10^3^/µL [IQR]	7.3 [3.3–11.9]	6.8 [2.8–12.6]	0.98 (0.91–1.05)	0.539
Platelet count, per 10^4^/µL [IQR]	12.5 [8.3–18.2]	12.5 [4.8–24.9]	0.99 (0.92–1.05)	0.698
Serum creatinine, per mg/dL [IQR]	0.78 [0.63–1.35]	0.76 [0.54–1.50]	1.07 (0.75–1.61)	0.680
CRP, per g/L, [IQR]	15.9 [1.4–57.2]	30.2 [1.80–71.9]	1.00 (0.99–1.01)	0.869
CRP < 100 mg/L	20 (91)	20 (80)	2.50 (0.48–18.9)	0.306
Indication of blood culture: fever	13 (59)	19 (76)	0.46 (0.12–1.57)	0.219
Fever during course	15 (68)	20 (80)	0.54 (0.12-2.00)	0.357
Persistent fever > 72 h	4 (18)	4 (16)	1.17 (0.24–5.59)	0.843
Causative organisms				
Gram positive	12 (55)	11 (44)	1.53 (0.48–4.93)	0.471
Gram negative	10 (45)	11 (44)	1.06 (0.33–3.39)	0.920
*Staphylococcus aureus*	7 (32)	3 (12)	3.42 (0.81–17.9)	0.109
*Escherichia coli*	2 (9)	6 (24)	0.32 (0.04–1.57)	0.190
*Klebsiella pneumoniae*	3 (14)	1 (4)	3.79 (0.44–80.1)	0.265
*Pseudomonas aeruginosa*	2 (9)	2 (8)	1.15 (0.13–10.3)	0.894
Coagulase-negative Staphylococci	1 (5)	3 (12)	0.35 (0.02–2.97)	0.378
*S. aureus* or *K. pneumoniae*	10 (45)	4 (16)	4.38 (1.19–18.9)	0.033*
Persistent BSI	7/15 (47)	2/14 (14)	5.37 (1.12–39.4)	0.053

IQR, interquartile range; anti-IL6R Ab, anti-interleukin-6 receptor antibody; PBS, Pitt bacteremia score; WBC, white blood cell; CRP, C-reactive protein; BSI, bloodstream infection

## Discussion

This study characterized the clinical features of BSI in patients receiving anti-IL6R Ab therapy and found a high prevalence (47%) of complicated BSI. Because IL-6 is a key mediator of CRP production and fever during the acute-phase response to infections [22, 23], the clinical presentations of infections are expected to be altered when IL-6 or its receptor is inhibited. Although atypical BSI presentations in anti-IL6R Ab recipients have been reported [7–10], data remain limited. We confirmed that the BSIs in anti-IL6R Ab recipients lacked classical features: fever was present in only 47% of episodes and CRP was normal in 30% at onset, and fever was absent throughout the course of illness in 26%. Consequently, one-third of blood cultures were obtained for the reasons other than fever. While these findings may be influenced by concomitant immunosuppression, the observed association between time from anti-IL6R Ab administration and serum CRP level suggests that anti-IL6R Ab itself suppresses CRP, as mechanistically expected. Despite atypical presentations, disease severity was substantial: sepsis and septic shock occurred in 47% and 26% of episodes, respectively, particularly among COVID-19 patients (45%), compared with 12% of rheumatic disease patients. In previous studies assessing the prevalence of serious infections among anti-IL6R recipients, reported incidence rates ranged from 1.0 to 7.0 per 100 patient-years, with some studies indicating that anti-IL6R Ab therapy is associated with an increased risk of serious infections comparing with tumor necrosis factor inhibitors (Table 4) [5, 24–27]. Septic shock was also linked to intra-abdominal infections, including perforative peritonitis, consistent with reports of tocilizumab use as a risk factor for diverticulitis [5, 6, 28]. In our study, the proportion of septic shock among sepsis cases was 55%, which is markedly higher than the 14.7% reported in a large epidemiological Japanese study [29]. Although this comparison should be interpreted with caution due to the differing definitions of sepsis between the studies, such a high proportion of septic shock has significant implications for patient care. Indeed, 30-day mortality rate in our study (19%) was slightly higher in that in the unselected patients with BSIs in a previous Japanese study [30]. Clinicians should therefore consider BSI in anti-IL6R Ab recipients with abnormal vital signs or symptoms suggestive of deep-seated infections, even in the absence of fever or CRP elevation, and also prepare for the patient’s rapid deterioration.

**Table 4:** A summary of the major studies assessing the incidence of serious/severe infections in anti-IL6R Ab recipients

Authors	Design	Drug	Number of patients included	Results
Campbell L et al.	systematic review and meta-analysis of 6 RCT	TCZ	3,102	ORs for serious infection were not significant between; 1) TCZ + MTX vs. MTX + placebo 2) TCZ monotherapy vs. MTX + placebo
Rutherford AI et al.	observational cohort	TCZ	2,174	Unadjusted IR of serious infection was 6.98/100PY in TCZ group, higher risk of serious infection comparing to etanercept (HR 1.22)
Pawar A et al.	multi-database cohort	TCZ	16,074	Tocilizumab was associated with an increased risk of serious bacterial infections (HR 1.19), skin and soft tissue infections (HR2.38), and diverticulitis (HR 2.34) versus TNFi
Fleischmann R et al.	8 clinical trials and their extensions	SAR	2,887	Unadjusted IR of serious infections were 3.7/100PY in combination therapy group and 1.0/100PY in monotherapy group

TCZ, tocilizumab; SAR, sarilumab; MTX, methotrexate; OR, odds ratio; IR, incidence rate; PY, patient-year; TNFi, tumor necrosis factor inhibitor

The distribution of causative organisms for BSIs among anti-IL6R Ab recipients were typical of those seen in the general population [31]. This suggests that anti-IL6R may not be associated with an increased risk of infection by specific pathogens. Additionally, no fungal BSIs were observed in our cohort. However, these findings may be attributable to the small sample size of this study. Further data collection is needed to clarify the impact of the anti-IL6R Ab on specific causative organisms.

Complicated BSI occurred in 47% of the episodes, affecting 52% of Gram-positive and 48% of Gram-negative BSIs. Notably, 70% (7/10) of *S. aureus* BSI were complicated, exceeding the previous reports: 43–60% for *S. aureus* BSI [15, 32], 34% for Gram-positive BSI [33] and 14% for Gram-negative BSI, which did not include infection-related mortality [16]. These findings suggest anti-IL6R Ab recipients are at high risk for complicated BSI. Recent studies support shorter antimicrobial courses for uncomplicated BSI [34–37], which may reduce adverse events and resistance [38]. However, complications of BSI such as IE, deep-seated abscesses, or bone and joint infections require surgical interventions or prolonged antimicrobial therapy. Our results highlight the need for active investigation of complications in anti-IL6R recipients with BSI.

Our study also revealed that community acquisition, underlying rheumatic diseases, BSI onset with 21 days of anti-IL6R Ab administration, BSI due to *S. aureus* or *K. pneumoniae*, and no additional immunosuppressive therapy were associated with complicated BSI in anti-IL6R Ab recipients, whereas high PBS (≥ 4 points) and higher pulse rate at presentation were inversely associated. In addition, although not statistically significant, febrile and lower systolic blood pressure at presentation appeared to be inversely associated with complicated BSI. Early predictors for complicated BSI are clinically important, as they can guide timely imaging studies and targeted investigations. Prior studies have described predictors for complicated BSI, including community-acquisition, skin examination findings suggesting acute systemic infection, persistent fever at 72 h, and positive follow-up blood culture in *S. aureus* BSI [15], as well as presence of indwelling prosthetic material, injection drug use, non-urinary source, BSI due to *S. marcescens*, *P. mirabilis* or *P. aeruginosa*, early clinical failure criteria, and persistent positive blood culture in Gram-negative BSI [16]. In addition, liver cirrhosis, diabetes mellitus, thrombocytopenia and high CRP level (> 100 mg/L) at bacteremic onset, delayed defervescence, and BSI-causing *K. pneumoniae* were identified as risk factors for abscess complication in community-acquired Enterabacterales BSI [39]. Our results – namely, community acquisition and *S. aureus* or *K. pneumoniae* etiology were associated with complicated BSI – are consistent with these previous studies, whereas persistent fever was not associated with complicated BSI. Interestingly, factors such as higher pulse rate and PBS ≥ 4 points were inversely associated with complicated BSI. These findings suggest that milder clinical presentations might hinder early recognition of BSI and consequently contribute to the development of complications. Moreover, BSI onset within 21 days of anti-IL6R Ab administration was another potential predictor, implying that immunodeficiency caused by anti-IL6R Ab may directly contribute the development of complicated BSI. However, these findings should be interpreted with caution given the retrospective design and relatively small sample size and potential biases. For instance, although not statistically significant, positive follow-up blood cultures appeared to be associated with complicated BSI. However, follow-up blood cultures were performed in 85% of Gram-positive BSIs, including all *S. aureus* cases, but in only one-third of Gram-negative BSIs. Interestingly, all five patients receiving no additional immunosuppressive agents other than anti-IL6R Ab developed complicated BSIs. All five episodes occurred in the rheumatic/MCD groups, four of which were community-onset. In the univariate analysis, additional immunosuppressive therapy was negatively associated with complicated BSI. Specifically, the receipt of cytotoxic chemotherapy or high-dose steroid therapy appeared to be negatively associated with complicated BSI, although this was not statically significant. However, these factors are associated with the COVID and CRS group and nosocomial onset of infection, which might confound their relationship with complicated BSI. At the same time, this finding may suggest that anti-IL6R Ab itself increase the risk of complicated BSIs.

Our study has several limitations. First, the relatively small number of BSI cases is a major limitation, and the small sample size—particularly the lack of multivariate analysis regarding the association with complicated BSI—limits the strength of our conclusions. Additionally, a case-control study incorporating a non-anti-IL6R Ab control group would provide a clearer assessment of the specific contribution of anti-IL6R Ab to BSI risk. Larger prospective studies with control groups are needed to better define early predictors of complicated BSI in anti-IL6R Ab recipients. Second, most patients received additional immunosuppressive agents alongside anti-IL6R Ab, including corticosteroids, immunosuppressants and cytotoxic chemotherapy. These agents represent significant confounders for BSI development. In particular, most cases in the CRS group had underlying hematological malignancies and received cytotoxic chemotherapy and lymphodepletion therapy, which strongly influence infection risk. Third, because there is no standardized consensus definition of complicated BSIs across different organisms, we adopted a modified definition based on criteria for *S. aureus* and GNR. Consequently, direct comparison our rates of complicated BSI with previous studies is methodologically limited. Finally, inflammatory markers other than CRP level, such as procalcitonin or erythrocyte sedimentation rate (ESR), might serve as predictive indicators for complicated BSI; however, these biomarkers were measured in only a small subset of cases and could not be evaluated.

In conclusion, we demonstrated that BSIs in anti-IL6R Ab recipients often lack typical signs of BSI such as fever and serum CRP level elevation, and 47% of episodes were complicated. Onset within 21 days of anti-IL6R Ab, underlying rheumatic disease, community-acquisition, and BSI caused by *S. aureus* or *K. pneumoniae* were associated with complicated BSI in univariate analysis, whereas persistent fever was not. These results underscore the importance of considering BSI in anti-IL6R Ab recipients even in the absence of classical features like fever or CRP elevation, and of performing thorough investigation for suppurative complications when blood cultures are positive.

## Data Availability

The data underlying this article will be shared on reasonable request to the corresponding author.
